# Nobiletin protected against hypertrophic cardiomyopathy via targeting PPARα

**DOI:** 10.3389/fphar.2025.1628625

**Published:** 2025-08-04

**Authors:** Kewei Zhou, Chang Chen, Hexin Cai, Zuqian Lian, Luping Wang, Qinghuo Li, Cancan Wang, Xiaoqian Wu, Panxia Wang

**Affiliations:** ^1^ School of Pharmaceutical Sciences, Guangzhou Medical University, Guangzhou, China; ^2^ Guangzhou Municipal and Guangdong Provincial Key Laboratory of Molecular Target & Clinical Pharmacology, The NMPA and State Key Laboratory of Respiratory Disease, Guangzhou Medical University, Guangzhou, China; ^3^ School of Foreign Languages, Guangzhou Medical University, Guangzhou, China

**Keywords:** *Citrus reticulata*, hypertrophic cardiomyopathy, nobiletin, naringenin, PPARα

## Abstract

**Background:**

Hypertrophic cardiomyopathy is an independent risk factor for heart failure. *Citrus reticulata* (*C. reticulata*) is a traditional Chinese medicine with a variety of chemical components and pharmacological effects. The mechanisms of *C. reticulata* for treating hypertrophic cardiomyopathy are still unclear.

**Methods:**

In this study, we used network pharmacology techniques combined with bioinformatics analysis and identified the active ingredient in *C. reticulata* to protect against hypertrophic cardiomyopathy. We analyzed the Gene Expression Omnibus (GEO) database from human heart tissue with hypertrophic cardiomyopathy to reveal the potential targets. Finally, molecular docking and *in vitro* validation were used to reveal the binding of the potential targets and the main active component of *C. reticulata*.

**Results:**

Our results revealed that there are five main active ingredients of *C. reticulata* (nobiletin, naringenin, sitosterol, 5,7-dihydroxy-2-(3-hydroxy-4-methoxyphenyl) chroman-4-one, and citromitin). By analyzing the intersecting genes between *C. reticulata* and hypertrophic cardiomyopathy, 40 hub genes were obtained. Gene ontology (GO) and Kyoto Encyclopedia of Genes and Genomes (KEGG) analysis indicated that the responses to oxidative stress and fatty acids were the main pathways for *C. reticulata* to act against hypertrophic cardiomyopathy. The protein–protein interaction analysis results showed that the main active ingredients of *C. reticulata* were nobiletin and naringenin, while peroxisome proliferator-activated receptors (PPAR)α might be the potential targets of *C. reticulata* in treating hypertrophic cardiomyopathy. The molecular docking results showed that the main active ingredient, nobiletin, could bind to PPARα with a strong hydrogen-bonding interaction force. *In vitro* results validated that nobiletin might directly bind to PPARα, thereby increasing the expression of lipid metabolism-related genes and relieving hypertrophic responses of cardiomyocytes.

**Conclusion:**

The nuclear receptor PPARα might be the potential endogenous receptor of the active ingredients of *C. reticulata*.

## 1 Introduction

Heart failure (HF) is the final stage of many cardiovascular diseases and remains a leading cause of death and disability worldwide (Angola et al., 2023). Hypertrophic cardiomyopathy (HCM) is a myocardial disease characterized by cardiac hypertrophy, with significant left ventricular hypertrophy accompanied by cardiac dysfunction (Murray, 2022; Zou et al., 2004; Bai et al., 2022). Cardiac hypertrophy is an independent risk factor for the progressive development of heart failure, which is characterized by increased protein synthesis and cardiomyocyte cell surface, and re-expression of fetal genes, such as atrial natriuretic peptide (ANF), brain natriuretic peptide (BNP), and β-myosin heavy chain (β-MHC) (Nakagawa, Nishikimi, and Kuwahara, 2019). The pathological mechanisms of hypertrophic cardiomyopathy are complex, involving abnormal activation or inhibition of multiple signaling pathways. Therefore, revealing the potential mechanisms and finding the potential target are critical for the treatment of hypertrophic cardiomyopathy.

As a high-energy-consuming organ, the heart needs a huge and continuous production of ATP to sustain the contractile function (Zou et al., 2004). The metabolic patterns of cardiomyocytes change with different conditions (Chen et al., 2021). Studies have shown that metabolic disorders and mitochondrial dysfunction are common pathogenic mechanisms in patients with hypertrophic cardiomyopathy, among which the most significant and noteworthy is the change in lipid metabolism (Ranjbarvaziri et al., 2021). Under physiological conditions, cardiomyocytes prefer to produce ATP via fatty acid oxidation (Da Dalt et al., 2023). Fatty acids are transported into cardiomyocytes, converted to fatty acyl-CoAs, converted to acyl-carnitine by carnitine palmitoyltransferase 1 (CPT1), and then transported into the mitochondria for β-oxidation to produce ATP (Grevengoed et al., 2014). In the heart of hypertrophic cardiomyopathy, there are abnormalities in the transport and utilization of free fatty acid (FFA) by cardiomyocytes due to the decrease in the content of lipid metabolism-related enzymes and acylcarnitine (Wang et al., 2022b; Previs et al., 2022; Ranjbarvaziri et al., 2021). When the oxygen supply is insufficient during cardiac hypertrophy, cardiomyocytes shift their energy metabolism from fatty acid oxidation to glycolysis (Huang et al., 2024). The metabolic pattern shifts to glycolysis during cardiac hypertrophy is only a compensatory way to rapidly meet the energy needs of cardiomyocytes (Chen et al., 2021). However, prolonged reliance on glycolysis as the main metabolic pathway is one of the critical reasons that cardiac hypertrophy proceeds to heart failure (Huang et al., 2024). Currently, improving cardiac lipid metabolism, oxidative stress, and mitochondrial damage is the main direction for treating hypertrophic cardiomyopathy (Sebastian et al., 2023). Therefore, targeting cardiac metabolic regulators holds the potential to stop or reverse the progression of HF (Noordali et al., 2018).

In recent years, traditional Chinese medicines and simple preparations have received more attention in the treatment of cardiovascular diseases (Gan et al., 2023; Koo et al., 2018). Many ancient books of traditional Chinese medicine have recorded various prescriptions for cardiovascular diseases. *Citrus reticulata* (*C. reticulata*) is one of the most productive fruit species and rich in citrus flavonoids. Flavonoids are the main active ingredients in *C*. *reticulata* and have various biological activities such as antioxidant, antiviral, anti-inflammatory, and anticancer activity (Wang et al., 2023). Previous studies have revealed that citrus flavonoids have great effects on preventing and treating cardiovascular diseases (Yu et al., 2018; Zou et al., 2022). Nobiletin, a polymethoxy flavonoid, protects against pressure-overload induced cardiac hypertrophy via inhibition of NADPH oxidases and endoplasmic reticulum stress (Zhang et al., 2017; Ke et al., 2023). Naringin, a citrus flavonone, protects cardiomyoblasts (H9C2 cells) against high glucose (HG)-induced apoptosis by modulating the activation of the p38 MAPK pathway (Han et al., 2009). However, it is still unclear whether citrus flavonoids regulate the metabolism of cardiomyocytes and play a role in cardiac hypertrophy.

In this study, we focused on the metabolism regulation of *C*. *reticulata* cardiomyocytes, used network pharmacology to analyze the key active ingredients during cardiac hypertrophy, and further validated the potential effects of *C*. *reticulata in vitro*. Our results showed that nobiletin, a key active ingredient of *C*. *reticulata*, improves myocardial lipid metabolism by directly targeting peroxisome proliferator-activated receptor alpha (PPARα) and inhibiting the phenylephrine (PE)-induced hypertrophic cardiomyopathy responses *in vitro*. This study investigated possible mechanisms using network pharmacology and validated them using cell experiments to elucidate the effects of *C*. *reticulata* in hypertrophic cardiomyopathy (HCM) treatment.

## 2 Materials and methods

### 2.1 Network pharmacology

#### 2.1.1 Screening of the main active composition of *Citrus reticulata* and prediction of potential targets

The Traditional Chinese Medicine Systematic Pharmacology Database and Analysis Platform (TCMSP, https://old.tcmsp-e.com/tcmsp.php) was used to obtain the absorption, distribution, metabolism, and excretion (ADME) properties of *C*. *reticulata* to predict the bioavailability and biological activity by using the keyword “*Citrus reticulata*.” First, the active ingredients of *C*. *reticulata* were identified based on the criteria of oral bioavailability (OB) ≥ 30% and drug likeness (DL) ≥ 0.18. Subsequently, the potential targets of these active ingredients in *C*. *reticulata* were identified using TCMSP. The obtained potential targets of *C*. *reticulata* were normalized by combining the STRING database (https://cn.string-db.org/) and the UniProt database (https://www.uniprot.org/).

#### 2.1.2 Potential targets of hypertrophic cardiomyopathy acquisition

The search term “hypertrophic cardiomyopathy” was entered into the GeneCards platform (https://www.genecards.org/) to search for all target genes under the standards of score ≥1.87 (median gene score). These acquired target genes were normalized and imported into the UniProt database (https://www.uniprot.org/) to obtain their UniProt ID. Duplicate targets were removed.

#### 2.1.3 The common targets of *Citrus reticulata* and hypertrophic cardiomyopathy

Subsequently, a Venn diagram was drawn to show the intersection targets of *C*. *reticulata* and hypertrophic cardiomyopathy by using the Jvenn website (https://jvenn.toulouse.inra.fr/app/index.html).

#### 2.1.4 Construction of an active composition–target–disease network

Cytoscape 3.10.1 was used to construct a network of active composition and the potential key targets of *C*. *reticulata* to treat hypertrophic cardiomyopathy. The nodes in the diagram represented active components (red), potential targets (yellow), hypertrophic cardiomyopathy (blue), and *C*. *reticulata* (blue). The network elucidates the interaction between the active components of *C*. *reticulata* and the potential targets against hypertrophic cardiomyopathy. More edges from the nodes represent more potential of active components or targets to affect hypertrophic cardiomyopathy.

#### 2.1.5 Construction of protein–protein interaction (PPI) network

The common targets of *C*. *reticulata* and hypertrophic cardiomyopathy were entered into the STRING database, which can predict PPIs. Cytoscape 3.10.1 (http://www.cytoscape.org/) was used to visualize the PPI network diagram. The node color was adjusted according to the degree in the diagram. The degree is correlated with the number of edges from each node and represents the strength of interactions between proteins. The deeper the color, the stronger the interactions.

#### 2.1.6 Gene ontology (GO) enrichment analysis and Kyoto Encyclopedia of Genes and Genomes (KEGG) pathway enrichment analysis

To understand the biological functions, the pathways, and localizations of these common genes enriched in the cells, GO enrichment analysis was performed to analyze cellular component (CC), molecular function (MF), and biological process (BP) items on massive genetic information. The KEGG enrichment analysis was used to systematically analyze the latest gene function annotations. The obtained common targets of *C*. *reticulata* and hypertrophic cardiomyopathy identified in Section 2.1.3 were imported into an online data analysis website, SRplot (https://www.bioinformatics.com.cn/srplot), to perform GO and KEGG enrichment analysis and produce bar and bubble graphs (Yin et al., 2023). The species parameter was set to “Human.” We selected the top 10 most significant GO terms and the top 10 KEGG pathways with the smallest *P* values.

#### 2.1.7 Gene Expression Omnibus (GEO) database analysis

To investigate the differentially expressed genes (DEGs) in heart tissue between patients with hypertrophic cardiomyopathy and normal individuals, we used the GEO database (https://www.ncbi.nlm.nih.gov/geo/) to obtain genomic data from patients and normal individuals. We searched and obtained heart tissue samples from eight patients with hypertrophic cardiomyopathy and five healthy donors in the GEO database using “hypertrophic cardiomyopathy” as the keyword (Series: GSE32453, GPL6104). The GEO2R tool (https://www.ncbi.nlm.nih.gov/geo/geo2r/) was used to gain the genetic data from patients and normal individuals and to obtain the DEGs related to hypertrophic cardiomyopathy with the restriction of |log_2_ (Fold Change)| > 2, and *P*-value < 0.05. The DEGs data were imported into OmicStudio (https://www.omicstudio.cn/) for principal component analysis (PCA). STRING and Cytoscape 3.10.1 were used to screen out the hub genes. Subsequently, the Science and Research online plot (SRplot) platform was used to analyze the cluster and expression of these hub genes. GO and KEGG pathway enrichment analysis were performed to obtain the differences in gene function between patients and normal individuals. The Jvenn website was used to obtain Venn maps of potential targets between drugs and hypertrophic cardiomyopathy.

#### 2.1.8 Molecular docking

The active composition structures of *C*. *reticulata* were downloaded from the PubChem database (https://pubchem.ncbi.nlm.nih.gov/), and ChemDraw software (https://revvitysignals.com/products/research/chemdraw) was used to convert the file format to *.SDF for the following docking analysis. The structures of potential target proteins (peroxisome proliferator-activated receptors (PPAR)α, PPARγ, and CREB) were downloaded from the RCSB Protein Data Bank (https://www.rcsb.org/) using the species as “*Homo sapiens*” and refinement resolution (Å) less than 2.0. Subsequently, the online Molecular Operating Environment (MOE) software (https://www.chemcomp.com/Products.htm) was used to perform molecular docking. Protein preparation was conducted with all bond orders reassigned, hydrogen atoms added, and water molecules deleted. Then, the energy minimization of the active components of *C*. *reticulata* hydrogens was conducted to realize the optimization of the hydrogen-bonding network. The docking analysis was conducted with the grid box containing the whole receptor using the “Rigid Receptor” docking method. Finally, 10 docking poses were exported, and molecular docking scores were obtained by the GBVI/WSAOG function to indicate the binding energy between the active composition of *C*. *reticulata* and the potential interactive target proteins. The validation of docking analysis was conducted using the same parameters.

The composite data file downloaded from the MOE with the highest docking score was imported into the Protein–Ligand Interaction Profiler (PLIP) (https://plip-tool.biotec.tu-dresden.de/plip-web/plip/index) to analyze the hydrogen bonds and non-covalent interactions between ligands and receptors. Finally, Pymol 2.6 software (http://pymol.org) was used to visualize the interaction between the active composition of *C*. *reticulata* and the potential targets.

#### 2.1.9 Molecular dynamics (MD) simulations

Molecular dynamics simulations were performed by using Yet Another Scientific Artificial Reality Application (YASARA) 10.3.16 (Krieger and Vriend, 2015) with the aid of the AMBER94 force field (Cornell et al., 1996). For the MD pre-processing, the receptor and ligand were separately imported into YASARA 10.3.16 to clean and optimize the structure and optimize the hydrogen bond networks. The complex model was used in a cubic simulation cell with a periodic boundary condition. The physiological conditions of the simulation cells were set as pH 7.4 and temperature 310 K. The energy minimization process was conducted by the simulated annealing method using the steepest gradient algorithms (Krieger et al., 2002). The time step of the simulation systems was set as 2.0 fs. The simulation trajectories were saved every 100 ps. The simulations were extended for 25,000 ps by following constant pressure and the Berendsen thermostat. The trajectories were utilized to analyze the root mean square deviations (RMSD) using “md_analyze.mcr” and root mean square fluctuations (RMSF) using “md_analyzeres.mcr.”

### 2.2 *In vitro* experimental verification

#### 2.2.1 Cell culture

H9C2 cardiac myoblasts were obtained from ATCC (Manassas, VA, United States) and cultured in Dulbecco’s modified Eagle’s medium (DMEM) (Gibco, United States) supplemented with 10% fetal bovine serum and incubated at 37°C and 5% CO_2_ at a density of 1 × 10^6^ cells per dish (35 mm). H9C2 cells were treated with PE (10 mM dissolved in dimethyl sulfoxide (DMSO) (TargetMol Molecule Corp., Boston, United States) for 24 h to mimic the *in vitro* hypertrophic cardiomyopathy model. Nobilietin (NOB), an active ingredient of *C*. *reticulata*, was purchased from TargetMol Molecule Corp. and dissolved in DMSO. NOB (20 mM) was administered into H9C2 cells prior to PE stimulation for 1 h.

#### 2.2.2 Total RNA extraction

Total RNA was extracted from H9C2 cells in different groups using TRIzol reagent according to the manufacturer’s instructions. RNA concentration and purity were determined using a NanoDrop One UltraMicro spectrophotometer (Thermo Fisher Scientific, Waltham, MA, United States). RNA samples with an OD260/OD280 ratio of 1.8–2.0 and an OD260/OD230 ratio of 2.0–2.4 were considered to have acceptable concentration and purity.

#### 2.2.3 Quantitative real-time PCR (qPCR)

One microgram of total RNA from each sample was reverse-transcribed to cDNA according to the EZB Color Reverse Transcription Kit (EZBioscience, Las Vegas, Clark, Nevada, United States). The expression of target genes was detected using the SYBR qPCR Master Mix kit on the Roche LightCycler^®^ 96 Instrument (F. Hoffmann-La Roche, Ltd., BASEL, CH). Primer information is provided in Table 1. The qPCR conditions and parameters were as follows: initial denaturation at 95°C for 30 s, followed by 40 cycles of amplification (95°C for 10 s and 60°C for 30 s). The gene cycle threshold (Ct) values were normalized, and relative quantitative values were extrapolated using the 2^−△△Ct^ method. β-actin served as an internal control.

**TABLE 1:** Primer sequence used for qPCR.

Gene name	Forward (5′ - 3′)	Rearward (5′ - 3′)
PPARα	GGC​TCT​GAA​CAT​TGG​CGT​TC	GAG​TTA​CGC​CCA​AAT​GCA​CC
CPT1A	AAA​CAG​ATC​TGC​CTG​TCG​GG	CAC​ACC​CAC​CAC​CAC​GAT​AA
ANF	GGG​AAG​TCA​ACC​CGT​CTC​AG	GAT​CTA​TCG​GAG​GGG​TCC​CA
BNP	GTG​CTG​CCC​CAG​ATG​ATT​CT	CGC​CGA​TCC​GGT​CTA​TCT​TC
β-actin	ACC​CTA​AGG​CCA​ACC​GTG​AA	ATG​CCA​GTG​GTA​CGA​CCA​GA

#### 2.2.4 Western blot

Total protein of cells was lysed by RIPA buffer (1×, containing 10 mM PMSF protease inhibitor) for 30 min on ice. The protein concentration was quantified with a BCA kit (Thermo, United States). Then, the sample with protein loading buffer (5×) was analyzed with SDS-PAGE, and the gels were transferred to polyvinylidene fluoride (PVDF) membranes (Millipore, United States). After blocking non-specific signals, the protein bands were incubated with primary antibodies at 4°C overnight. The following antibodies were used: PPARα (66826-1-1g, Proteintech Group, Inc., IL, United States, 1:1,000) and GAPDH (60004-1-Ig, Proteintech, 1:50,000). Afterward, secondary antibodies were incubated at room temperature for 1 h, and the blots were visualized with an ECL kit (Tanon, Shanghai, China) using a chemical image system (Amersham Imager 600, United States). The band intensity was analyzed by ImageJ software.

#### 2.2.5 Cellular thermal shift assay (CETSA)

As described previously (Zhang et al., 2022), control cells were incubated with the same volume of PBS. Cells were cultivated and counted, followed by resuspension in PBS (containing 1 mmol/L PMSF) to a final density of 2 × 10^7^/mL. Cells were sub-packaged into seven PCR tubes and heated with a thermal gradient from 37°C to 67°C for 3 min. After freeze–thawing twice with liquid nitrogen, the supernatant was separated by centrifugation at 12,000 g for 25 min and collected. A 20-μL aliquot of the supernatant was loaded onto an SDS-PAGE gel, followed by Western blotting. CETSA curve analysis was performed using GraphPad Prism software 9.0.0 (GraphPad Software Inc., San Diego, CA, United States).

#### 2.2.6 Measurement of the cardiomyocyte surface area

H9C2 cells were seeded into a 6-well plate at a density of 8 × 10^5^, fixed with 4% (w/v) paraformaldehyde for 15 min, and then treated with 0.3% (v/v) Triton X-100 for 15 min at room temperature to permeabilize the membrane. After washing with phosphate-buffered saline (PBS) three times, the cells were stained with 0.1% rhodamine–phalloidin (Yeasen, Wuhan, China) for 1 h at room temperature to visualize the actin filaments of cardiomyocytes. Then, the cells were washed with PBS three times, and 4, 6-diamidino-2-phenylindole (DAPI, Invitrogen, #D1306, CA, United States) was used to indicate the nucleus. Images from six fields of each group were captured by a Nikon Ti2-E inverted fluorescence microscope (Nikon, Japan). The surface area from 100 to 200 cells was measured by ImageJ software, which was blinded to observers.

#### 2.2.7 Mitochondrial superoxide generation assay

H9C2 cells were seeded into a 6-well plate at a density of 8 × 10^5^. The cells were washed with PBS three times, fixed with MitoSOX RED (MA, United States, Thermo) 5 μM (dissolved in Serum-Free DMEM), and then incubated for 30 min at 37°C and 5% CO_2_. The cells were gently washed three times with warm buffer. Images of each group were captured by a Nikon Ti2-E inverted fluorescence microscope (Nikon, Japan). The fluorescence intensity was measured by ImageJ software, which was blinded to observers.

#### 2.2.8 Statistical analysis

Data were analyzed by using GraphPad Prism 9 software (San Diego, CA, United States) and presented as mean ± SD. The Shapiro–Wilk test was used to test whether the data conformed to a normal distribution. Difference analysis between the two groups was performed by Student’s t-test. Difference analysis among various groups was performed by one-way ANOVA with Tukey’s *post hoc* test. Data were considered statistically significant with a *P* value less than 0.05.

## 3 Result

### 3.1 Active compositions and the potential targets of *Citrus reticulata*

In the TCMSP database, “*Citrus reticulata*” was used as the keyword to search for the various chemical components in *C*. *reticulata*. A total of five effective active compounds of *C*. *reticulata* were identified based on the limitation of OB ≥ 30% and DL ≥ 0.18. They are sitosterol, naringenin, 5,7-dihydroxy-2-(3-hydroxy-4-methoxyphenyl) chroman-4-one, citromitin, and nobiletin (details are shown in Table 2). Subsequently, the molecular IDs of these five main active compositions were imported into the TCMSP database to obtain the potential targets of *C*. *reticulata*. These targets were imported into the STRING database and the UniProt database to obtain the standard gene symbols. After removing duplicate targets, 66 potential targets of *C*. *reticulata* active ingredients were obtained (Supplementary Table S1).

**TABLE 2:** Main active ingredients in *Citrus reticulata*.

Molecular ID	Compound	OB (%)	DL
MOL000359	Sitosterol	36.91	0.22
MOL004328	Naringenin	59.29	0.4
MOL005100	5,7-Dihydroxy-2-(3-hydroxy-4-methoxyphenyl) chroman-4-one	47.74	0.31
MOL005815	Citromitin	86.9	0.14
MOL005828	Nobiletin	61.67	0.13

### 3.2 Prediction of hypertrophic cardiomyopathy targets

The GeneCards database was used to search for confirmed or potential targets of hypertrophic cardiomyopathy with “hypertrophic cardiomyopathy” as the keyword. A total of 4,336 genes related to hypertrophic cardiomyopathy were obtained. Based on a median gene score of 1.87, a total of 2,168 genes with a score ≥1.87 were identified.

### 3.3 *Citrus reticulata* active composition–target disease network diagram

**FIGURE 1:**
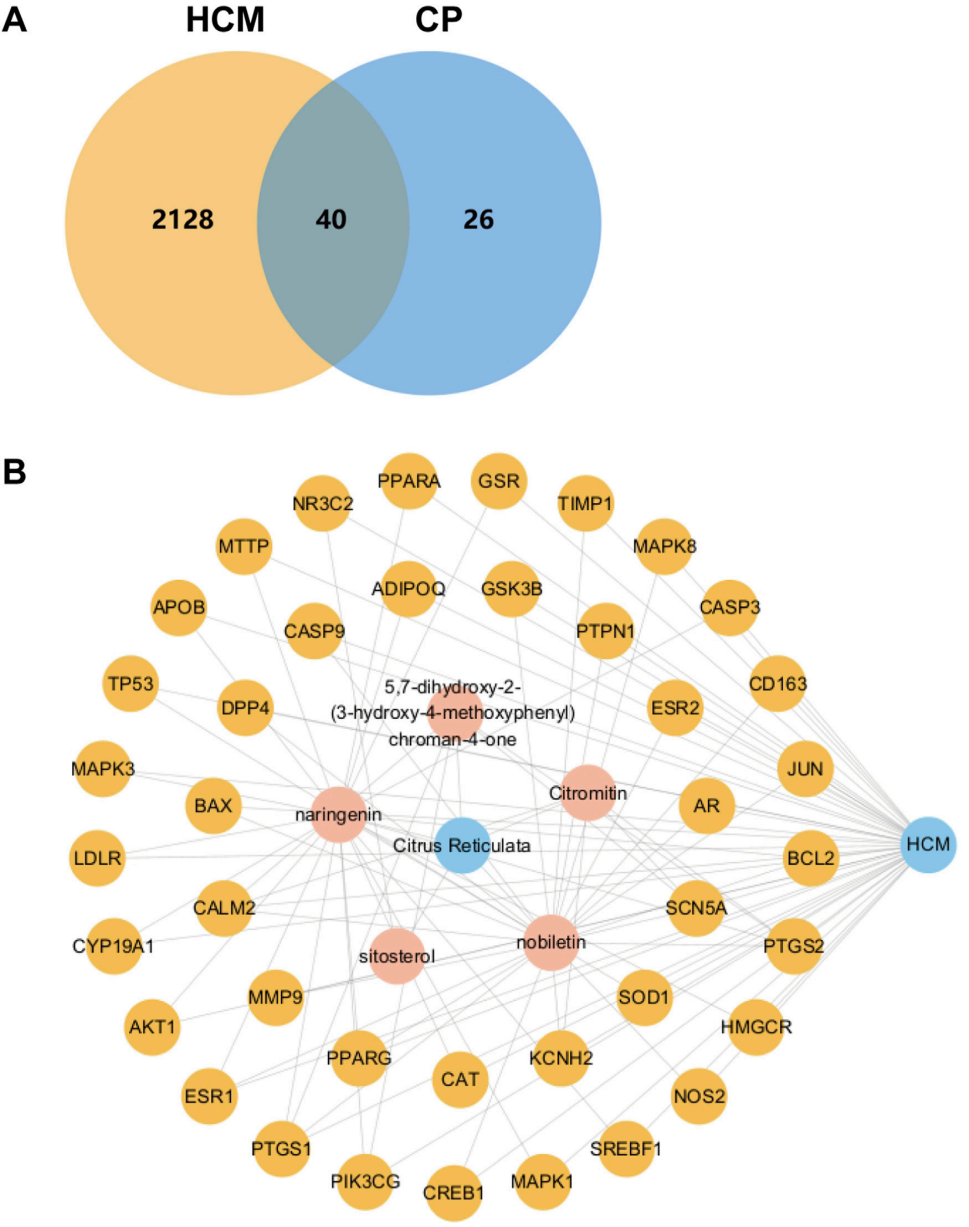
Venn diagram and active ingredient–target disease network. **(A)** Venn diagram of active ingredients of *C*. *reticulata* and the potential disease targets. **(B)** The relationship network diagram was drawn between potential therapeutic targets against hypertrophic cardiomyopathy and active components of *C*. *reticulata*. HCM, hypertrophic cardiomyopathy; CP, *C*. *reticulata*.

The potential targets of active ingredients in *C*. *reticulata* and the total potential 2,168 disease-related genes were imported into the Jvenn website to create a Venn diagram (Figure 1A). There are 40 intersecting potential target genes of *C*. *reticulata* and hypertrophic cardiomyopathy (Figure 1A). A relationship network was built between potential targets related to hypertrophic cardiomyopathy and *C*. *reticulata* active compositions (Figure 1B). Cytoscape 3.10.1 software was used to analyze the node degree values. There are 47 nodes and 198 edges in the network. The average degree of the network is 4.21, and there are five active component nodes of *C*. *reticulata* and 40 target nodes larger than this value. Among them, the active components with a high number of connected targets in *C*. *reticulata* were nobiletin (degree = 24) and naringenin (degree = 22). Based on the above results, it was concluded that nobiletin has the highest degree of active ingredients of *C*. *reticulata*, followed by naringenin.

In addition, the targets connected more active components included peroxisome proliferator-activated receptor alpha (PPARα) (degree = 2), peroxisome proliferator-activated receptor gamma (PPARγ) (degree = 2), and cyclic AMP-responsive element-binding protein 1 (CREB1) (degree = 2). The interaction of these five active ingredients with the targets illustrates the synergistic therapeutic effect of *C*. *reticulata* with multiple ingredients and multiple targets.

### 3.4 The PPI network of *Citrus reticulata* and hypertrophic cardiomyopathy common targets

**FIGURE 2:**
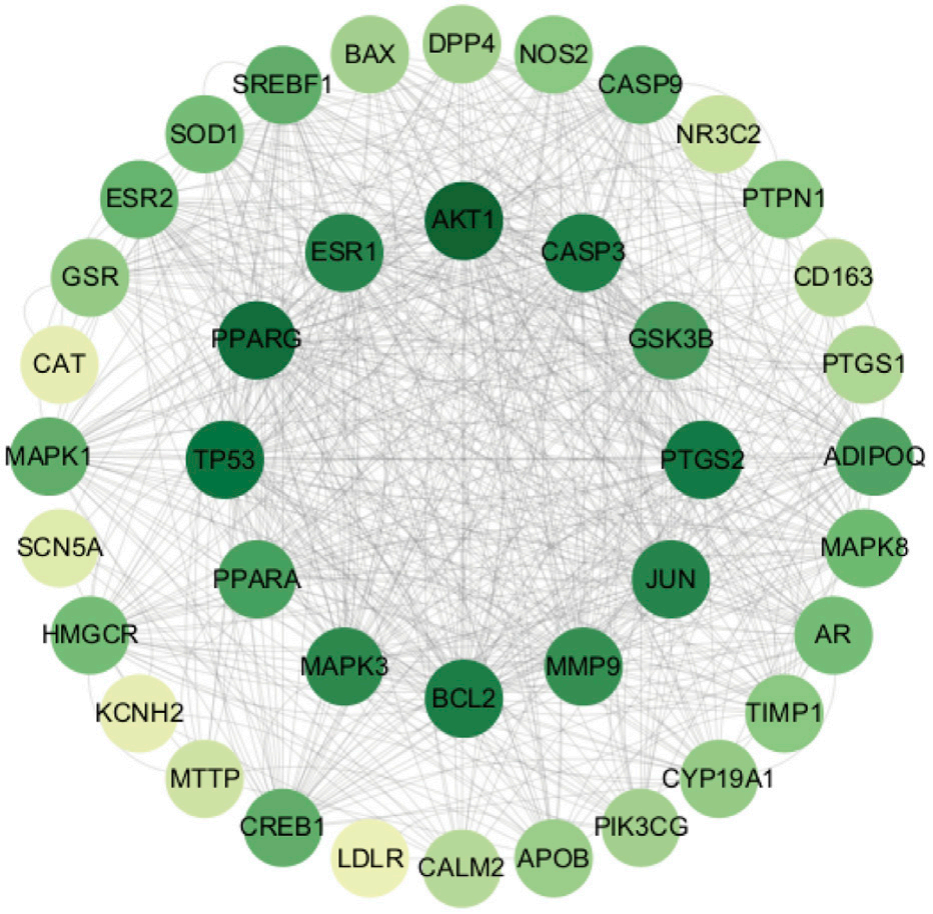
Protein–protein interaction network of *Citrus reticulata* against hypertrophic cardiomyopathy.

The STRING platform and Cytoscape 3.10.1 software were used to visualize the PPI network diagram of *C*. *reticulata* for hypertrophic cardiomyopathy. The result (Figure 2) shows that there are 40 nodes and 1,376 edges in the network. The node degree average is 34.4, and there are 19 targets exceeding the average. The top 20 nodes are as follows: AKT1, PPARγ, TP53, PTGS2, BCL2, CASP3, ESR1, JUN, MAPK3, MMP9, GSK3B, PPARα, ADIPOQ, CASP9, CREB1, MAPK1, SREBF1, ESR2, MAPK8, and AR (the node details in Table 3).

**TABLE 3:** Information about the top 20 genes of PPI.

Target	Degree	Target	Degree
AKT1	68	GSK3B	48
PPARγ	64	PPARα	46
TP53	62	ADIPOQ	44
PTGS2	60	CASP9	40
BCL2	58	CREB1	40
CASP3	58	MAPK1	40
ESR1	56	SREBF1	40
JUN	56	ESR2	38
MAPK3	54	MAPK8	36
MMP9	52	AR	34

### 3.5 GO analysis and KEGG pathway enrichment analysis

**FIGURE 3:**
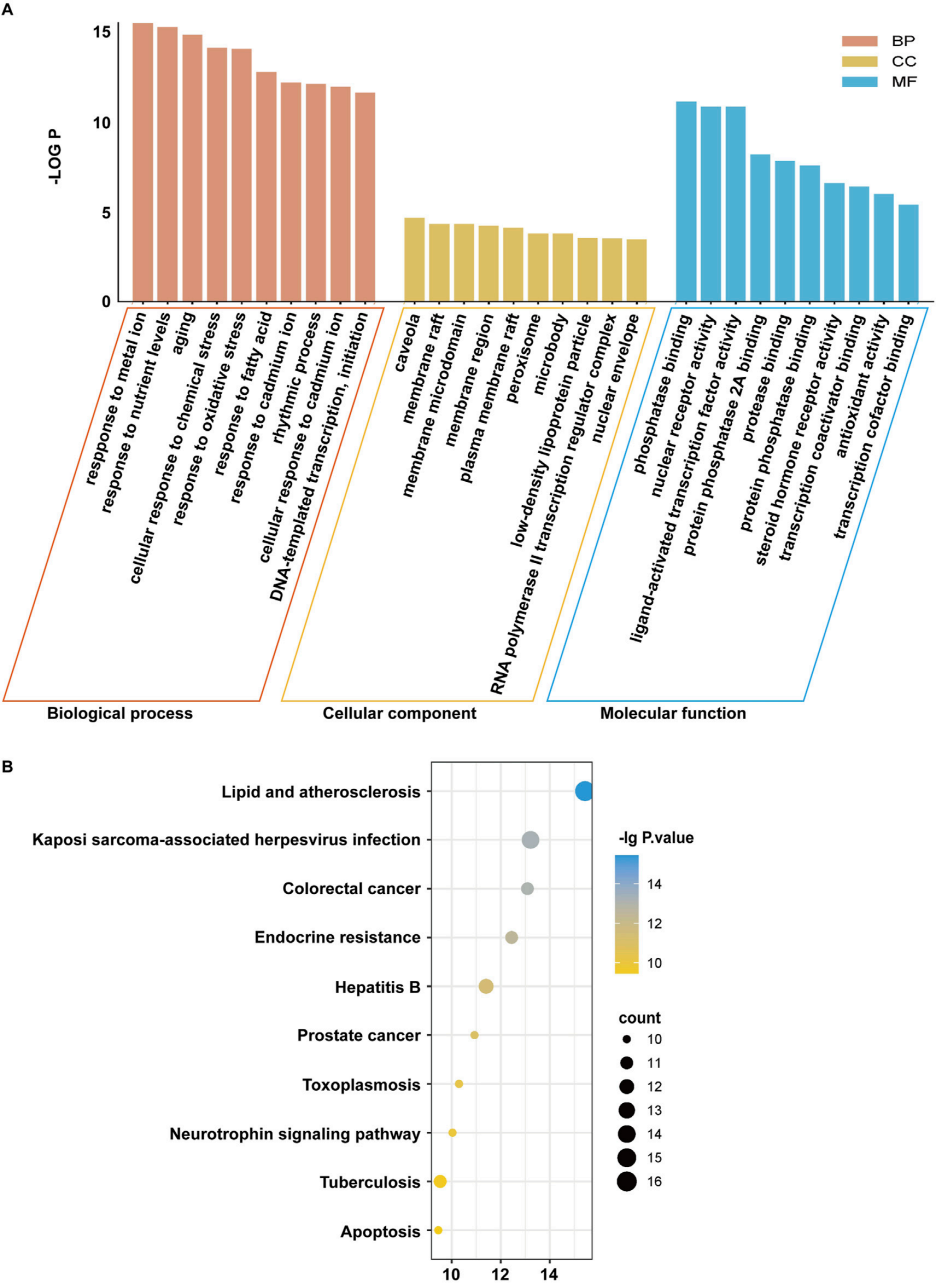
The GO enrichment and KEGG pathway analyses of *Citrus reticulata* active ingredients targets. **(A)** GO enrichment results. **(B)** KEGG enrichment results.

The enrichment analyses of biological processes (BP), cellular components (CC), molecular function (MF), and KEGG pathway were performed on the 40 potential targets in Figure 1A. The SRplot platform was used to analyze the enrichment. Our results showed that the numbers of CC, MF, and BP are 67, 139, and 1,650, respectively (*P* < 0.05). The results involved processes such as “Response to oxidative stress,” “Response to fatty acids,” “DNA template transcription and initiation,” and “apoptosis.” The main cellular component involved is the plasma membrane. The molecular functions of gene products mainly included phosphatase binding, nuclear receptor activity, and ligand-activated transcription factor activity (Figure 3A). In the KEGG pathway analysis, the results demonstrated that the lipid and atherosclerosis and the endocrine resistance pathways were the key pathways (Figure 3B).

Based on the GO and KEGG enrichment analysis results, it was speculated that the active compositions of *C*. *reticulata* might regulate lipid metabolism and oxidative stress processes, thereby protecting against hypertrophic cardiomyopathy. The PPAR pathway involving PPARα and PPARγ is closely related to lipid metabolism (Mirza et al., 2019; Yin et al., 2006; Montminy et al., 1990), and CREB1, as a transcription factor, is involved in DNA transcription and initiation (Ramakrishnan and Pace, 2011). Therefore, PPARα, PPARγ, and CREB1 might be the potential target genes of *C*. *reticulata*.

### 3.6 Acquisition of differentially expressed genes during hypertrophic cardiomyopathy

**FIGURE 4:**
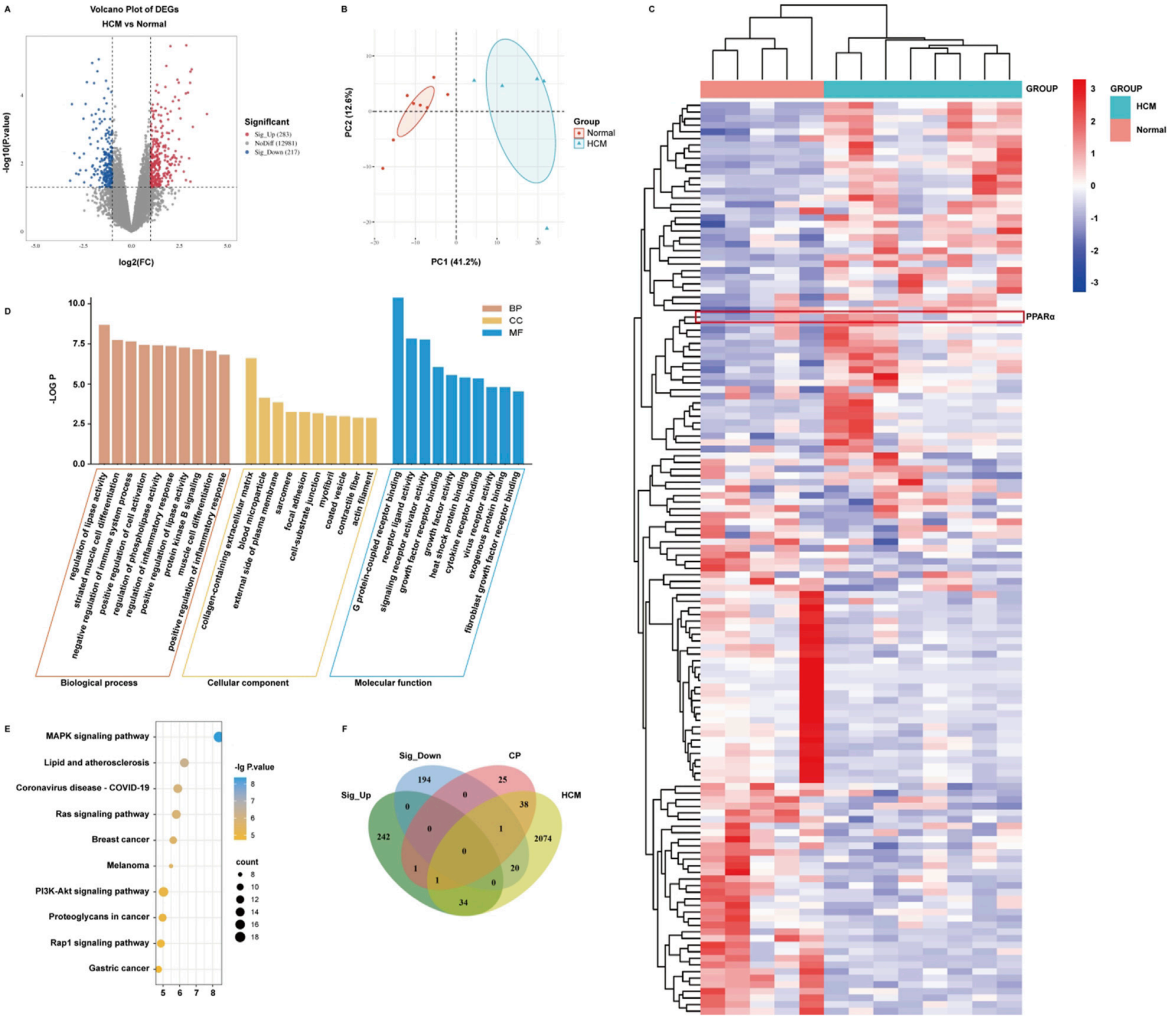
PPARα expression is significantly elevated in the heart of patients with hypertrophic cardiomyopathy. **(A)** Significance analysis of sample genes. The red part indicates that the gene expression is significantly upregulated compared to normal individuals. The blue part indicates that the gene expression is significantly downregulated compared to normal individuals. The gray part indicates that there is no difference in gene expression compared to normal individuals. **(B)** Verifying the differences between the two sets of data. **(C)** Taking DEGs to clustering analysis. **(D,E)** Importing DEGs to conduct GO and KEGG enrichment analysis. **(F)** Taking DEGs, target genes of *C*. *reticulata,* and disease target genes to conduct an intersection analysis.

To investigate the genetic differences in myocardial tissue between hypertrophic cardiomyopathy patients and normal individuals in clinical practice, we searched the GEO database using “HCM” as a keyword. Gene data in these samples were obtained by using the GEO2R platform, and the DEGs were analyzed by using the OmicStudio platform with the limitation of |log_2_ (Fold Change)| > 2 and *P* value < 0.05. A total of 500 DEGs were obtained, including 283 upregulated and 217 downregulated (Figure 4A; Supplementary Tables S2, S3). A volcano plot (Figure 4A), a PCA plot (Figure 4B), and a heatmap (Figure 4C) were generated to show the distribution of DEGs. Subsequently, the DEGs were imported into the SRplot platform for GO and KEGG enrichment analyses. The results showed that the DEGs focused on biological processes such as regulation of lipase activity, regulation of phospholipase activity, and protein kinase B signaling (Figure 4D). The main cellular components involved are concentrated in the collagen-containing extracellular matrix and the external side of the plasma membrane (Figure 4D). The molecular functions of gene products are concentrated in G protein-coupled receptor binding, receptor–ligand activity, and signal receptor activator activity (Figure 4D). The KEGG enrichment analysis results show that the action pathways involved by DEGs are mainly lipid and atherosclerosis, the Ras signaling pathway, and the MAPK signaling pathway (Figure 4E). Furthermore, we identified the core potential genes of *C*. *reticulata* treatment for hypertrophic cardiomyopathy and obtained two overlapping genes (Figure 4F). Interestingly, PPARα was one of the overlapping genes, which was consistent with the potential targets of *C*. *reticulata* related to hypertrophic cardiomyopathy (Figures 1B, 4F).

### 3.7 Molecular docking and molecular dynamics simulation

**FIGURE 5:**
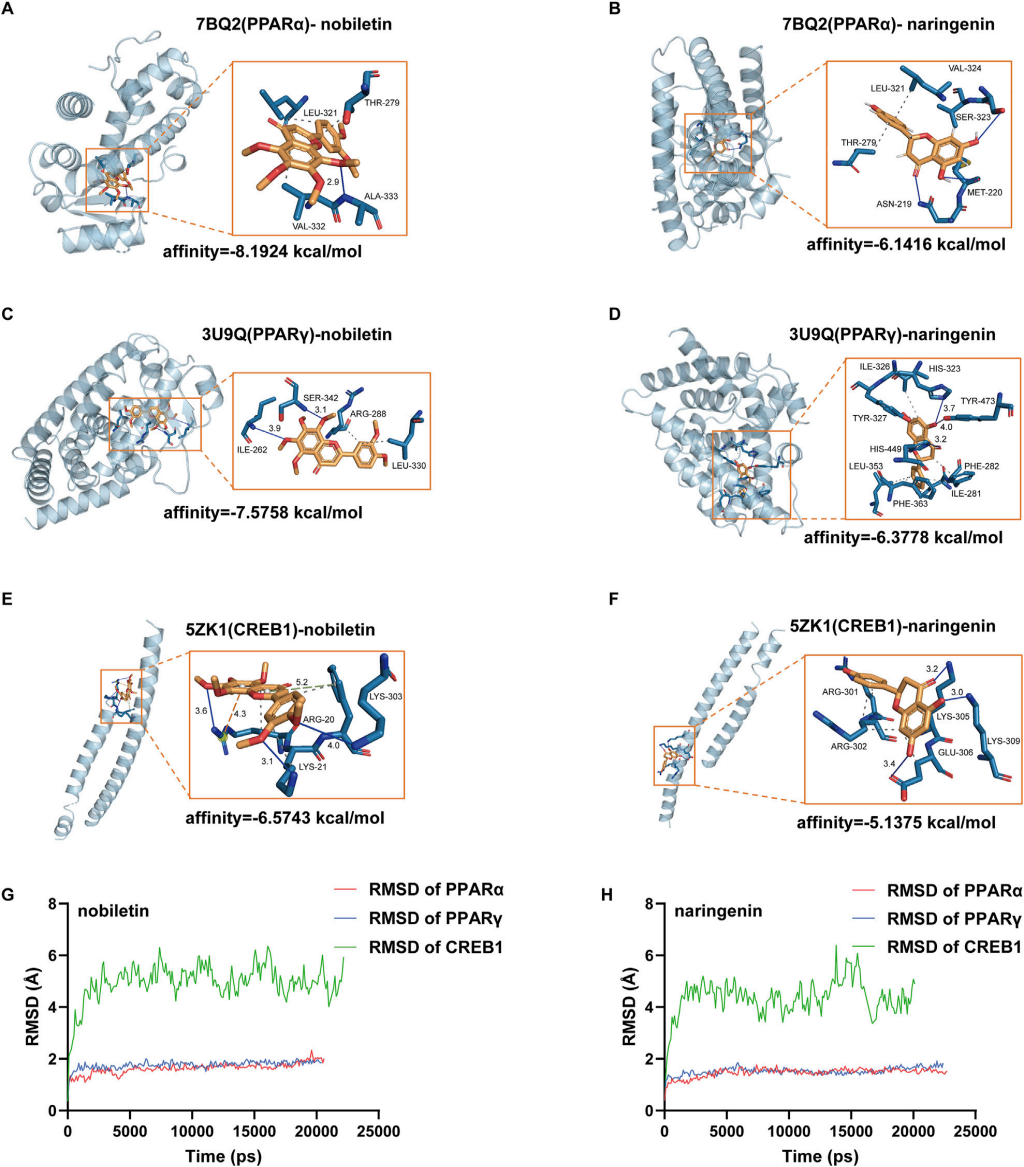
Molecular docking maps of hub targets with active compounds. **(A)** Docking result between PPARα and nobiletin. **(B)** Docking result between PPARα and naringenin. **(C)** Docking result between PPARγ and nobiletin. **(D)** Docking result between PPARγ and naringenin. **(E)** Docking result between CREB1 and nobiletin. **(F)** Docking result between CREB1 and naringenin. Blue represents hydrogen bonds; the gray dotted line represents hydrophobic interaction; the orange dotted line represents the π–cation interaction. **(G)** RMSD of nobiletin binding with PPARα, PPARγ, and CREB1. **(H)** Naringenin binding with PPARα, PPARγ, and CREB1. RMSD, root mean square deviation.

We subjected the hub genes of *C*. *reticulata* (PPARα, PPARγ, and CREB1) and the two most active ingredients of *C*. *reticulata* (nobiletin and naringenin) to molecular docking analysis by using MOE software. The binding energy of active ingredients of *C*. *reticulata* with hub genes is shown in Table 4. The result indicated that the complexes PPARα–nobiletin, PPARα–naringenin, PPARγ–nobiletin, PPARγ–naringenin, CREB1–nobiletin, and CREB1–naringenin, have good binding affinities (Table 4). Finally, PyMol software was used to visualize the molecular docking graphs (Figure 5). The more stable the ligand-receptor binding, the lower the binding energy will be. PPARα bound to nobiletin with the lowest energy (Figure 5A). The PLIP was used to obtain specific information on the hydrogen bonds formed between PPARα and the active ingredients, where “AA” is an amino acid with functional relationships, “D-A” is the distance between ligands and amino acids, and “angle” is the bond angle (Table 5; Supplementary Tables S4–S7).

**TABLE 4:** Binding energy of hub genes with active ingredients (kcal·mol^−1^).

Gene name	PDB ID	Binding energy	
		Nobiletin	Naringenin
PPARα	7BQ2	−8.1924	−6.1416
PPARγ	3U9Q	−7.5758	−6.3778
CREB1	5ZK1	−6.5743	−5.1375

**TABLE 5:** Hydrogen bonds of PPARα with the main active ingredients of *Citrus reticulata* from docking analysis.

Protein	PPARα			
Ligand	Type	AA	D‐A (Å)	Angle (°)
Nobiletin	H-BOND	ALA-333	2.91	147.94
Naringenin	H-BOND	SER-323	3.67	120.31
	H-BOND	MET-220	3.83	106.45
	H-BOND	ASN-219	3.23	124.55

To evaluate the stability of the ligand–target complex over time, we performed molecular dynamics simulations using YASARA 10.3.16. The RMSD and RMSF results of six ligand–receptor complexes were analyzed. RMSD measures the deviation of a structure at a given time point from its initial conformation and was used to assess the temporal stability of the ligand–target complexes. The results revealed that the systems of nobiletin bound to PPARα and PPARγ gradually stabilized after 200 ps and remained stable, showing similar trends without significant fluctuations (Figure 5G). In contrast, the system of nobiletin bound to CREB1 exhibited poor stability with high fluctuation levels. The naringenin complexes with PPARα, PPARγ, and CREB1 displayed similar stability trends (Figure 5H). Furthermore, the RMSF analysis results in Supplementary Figure S2 combined with molecular docking result (Figure 5A) revealed that the binding of nobiletin to PPARα involved higher RMSF values at THR-279, LEU-321, ALA-333, and VAL-332, indicating greater flexibility at these amino acid positions that may facilitate binding interactions. Altogether, our results show that PPARα had a better affinity with nobiletin, the main active ingredient of *C*. *reticulata*. All these results indicated that PPARα might be the hub gene and mediate the protective effects of *C*. *reticulata* against hypertrophic cardiomyopathy.

### 3.8 The main active ingredient of *Citrus reticulata* relieved cardiomyocyte hypertrophy and reactive oxygen species (ROS) via targeting PPARα

**FIGURE 6:**
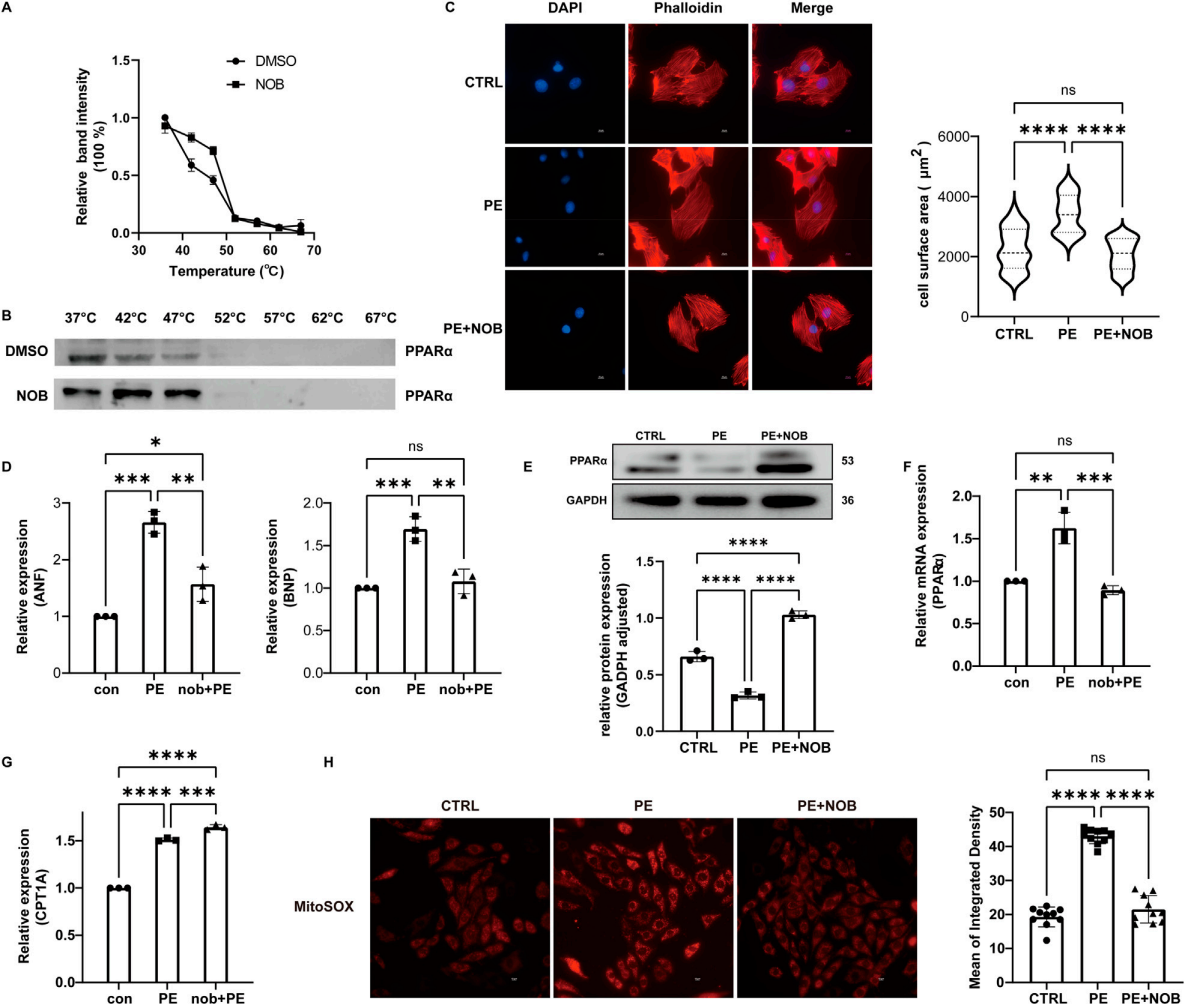
Nobiletin has a good binding effect with PPARα and has a significant therapeutic effect on cell hypertrophy. **(A,B)** CETSA result. **(C)** Fluorescence staining observation of the cell surface area and statistical analysis of the cell surface area. Scale bar: 10 μm. **(D)** Relative expression of ANF and BNP. **(E)** The protein expression of PPARα was assayed by WB. **(F)** The mRNA level of PPARα was assayed by qPCR. **(G)** Relative mRNA of CPT1A. **(H)** Result of mitochondrial superoxide generation assay. NOB, nobiletin; ANF, atrial natriuretic peptide; BNP, brain natriuretic peptide.

To validate the potential target of *C*. *reticulata*, we performed a cellular thermal shift assay (CETSA) to investigate the binding of nobiletin with PPARα. The CETSA results from H9C2 cells showed that nobiletin largely improved the thermal stability of PPARα (Figures 6A,B), which indicated that the active ingredients of *C*. *reticulata* could directly interact with PPARα. Therefore, we investigate the molecular mechanisms of the active ingredients of *C*. *reticulata* against hypertrophic cardiomyopathy. Our results showed that PE significantly induced the enlargement of the cell surface area of cardiomyocytes (Figure 6C) and the increased expressions of ANF and BNP (Figure 6D), which was notably alleviated with nobiletin treatment (Figures 6C,D). We measured the expression of PPARα and the target genes that are closely related to fatty acid metabolism. Our result showed that PE stimulation significantly inhibited the protein and the mRNA levels of PPARα (Figures 6E,F), while nobiletin treatment significantly augmented the expression of PPARα. Consistently, the mRNA of CPT1A, a downstream target gene of PPAR, was also increased by nobiletin treatment during PE stimulation (Figure 6G). In addition, we assayed the production of mitochondrial superoxide in cells. The results showed that the production of mitochondrial superoxide in the PE treatment group significantly increased, indicating that the cells had obvious oxidative stress (Figure 6H). However, the oxidative stress of cells in the nobiletin treatment group was significantly improved (Figure 6H). These results indicated that the active ingredient of *C*. *reticulata* could directly target PPARα and ameliorate oxidative stress to ameliorate hypertrophic cardiomyopathy.

## 4 Discussion

Hypertrophic cardiomyopathy is a relatively common inherited cardiac condition with potential for sudden cardiac death that affects 0.2% of the population (Dungu et al., 2024). The HCM clinical phenotype is characterized by asymmetric hypertrophy and diastolic dysfunction, with preserved or even slightly increased left ventricular ejection fraction (Wijnker et al., 2024). At the cellular level, hypertrophic cardiomyopathy is associated with myocyte hypertrophy, and the progressive disease leads to myocardial fibrosis and heart failure (Schlittler et al., 2023). However, treatment to prevent hypertrophic cardiomyopathy-induced cardiac dysfunction is lacking. Consequently, it is necessary to identify the hub genes and reveal the potential compounds to improve the clinical outcomes of hypertrophic cardiomyopathy.

TCM is also widely used as a medical treatment for hypertrophic cardiomyopathy (Wan et al., 2023). *C*. *reticulata* is a widely used traditional Chinese medicine with abundant resources worldwide (Shorbagi et al., 2022). As a multi-efficacy pericarp, *C*. *reticulata* is historically used to treat cardiovascular diseases (Zou et al., 2022). There are approximately 140 chemical ingredients in *C*. *reticulata* (Yu et al., 2018). Therefore, the potential targets and mechanisms of *C*. *reticulata* are complex, which challenges the research and development of TCM on cardiovascular diseases.

In the present study, we identified five main active ingredients in *C*. *reticulata* with 40 potential target genes. Based on our results, compounds nobiletin (degree = 24) and naringenin (degree = 22) might be the most active ingredients in *C*. *reticulata* with higher degree values to treat hypertrophic cardiomyopathy. Previous studies have revealed that flavonoids were regarded as the most important bioactive ingredients in *C*. *reticulata* (Liu et al., 2013). Both nobiletin and naringenin are citrus fruit-derived flavonoids that possess significant biological activity, including anticancer and anti-inflammatory effects (Zhou et al., 2021; Yahya and Al-Shawi, 2024). Nobiletin attenuates pathological cardiac remodeling following acute myocardial infarction via restoring autophagy flux (Wu et al., 2017). *In vivo* and *in vitro* experiments demonstrated that naringenin also improved cardiomyocyte function in sepsis-induced myocardial dysfunction via its anti-inflammatory and antioxidant properties (Pan et al., 2024). Both nobiletin and naringenin play a pivotal role in treating various cardiovascular diseases. In this study, our PPI network results revealed that PPARα, PPARγ, and CREB1 were the potential targets of *C*. *reticulata* with higher relevance for combating hypertrophic cardiomyopathy. Peroxisome proliferator-activated receptors (PPARs) are a three-membered subfamily of the nuclear receptor, including PPARα, PPARβ/δ, and PPARγ (Fougerat et al., 2024). PPARα is mainly expressed in cells with abundant mitochondria and higher energy demand, such as the heart, liver, and kidney (Jain et al., 1998). PPARβ has an ubiquitous expression in all cell types, while PPARγ is predominantly expressed in adipose tissue (Rubio-Ruíz et al., 2024). As nuclear regulatory factors, PPARs transcriptionally regulate the expression of many genes and fine-tune metabolic, inflammatory, and fibrotic processes. Previous studies have shown that PPARα and PPARγ are physiological master switches in the heart that regulate lipid metabolism and mitochondrial function (Legchenko et al., 2018; Wang et al., 2021). As a nuclear receptor, PPAR transcriptional activity depends on ligand activation. It is believed that the diversity of PPAR functions mostly depends on the variety of ligands. Therefore, identifying the natural compounds that directly regulate PPARs might be an effective strategy to improve cardiac function.

In this study, we reveal that the bioactive ingredients of *C*. *reticulata* potentially target PPARα and PPARβ to alleviate hypertrophic cardiomyopathy. A previous report has revealed that nobiletin attenuated lipid accumulation and the extent of atherosclerotic lesions and further alleviated atherosclerosis by inhibiting lipid uptake via the PPARγ/CD36 pathway (Wang et al., 2022a). By upregulating the expression of PPARα and PPARγ, naringenin also effectively inhibited cardiomyocyte hypertrophy in diabetic conditions (Zhang et al., 2019). In this study, the molecular docking results showed that both PPARα and PPARβ exhibited good binding affinities with the bioactive ingredients of *C*. *reticulata*. Moreover, PPARα demonstrated more specific hydrogen bonds. In combination with the GEO analysis, PPARα was identified as the hub gene of *C*. *reticulata*. However, the sample size of the GEO database used in this manuscript was small, which might be one limitation of this study. Our *in vitro* results validated the anti-hypertrophic responses of nobiletin and revealed that nobiletin, one of the active ingredients of *C*. *reticulata*, could directly interact with PPARα, which increased the expression of the downstream target genes.

## 5 Conclusion

In this study, we used network pharmacology techniques combined with bioinformatics analysis and identified the main active ingredients of *C*. *reticulata* that protect against hypertrophic cardiomyopathy. Our results revealed that nobiletin and naringenin are the main bioactive ingredients against hypertrophic cardiomyopathy. The nuclear receptor PPARα might be the endogenous receptor of the active ingredients of *C*. *reticulata*. By directly targeting PPARα, the active ingredients of *C*. *reticulata* increased the expression of lipid metabolism-related genes and relieved hypertrophic responses of cardiomyocytes.

## Data Availability

The datasets presented in this study can be found in online repositories. The names of the repository/repositories and accession number(s) can be found in the article/Supplementary Material.

## Glossary

ADME: Absorption, distribution, metabolism, and excretion
ANF: Atrial natriuretic factor
BNP: Brain natriuretic peptide
BP: Biological process
CC: Cellular component
CETSA: Cellular thermal shift assay
CPT1: Carnitine palmitoyltransferase 1
CREB1: Cyclic AMP-responsive element-binding protein 1
DEG: Differentially expressed genes
DL: Drug likeness
FFA: Free fatty acid
DMEM: Dulbecco’s modified Eagle’s medium
DMSO: Dimethyl sulfoxide
GO: Gene ontology
HF: Heart failure
HCM: Hypertrophic cardiomyopathy
HG: High glucose
KEGG: Kyoto Encyclopedia of Genes and Genomes
GEO: Gene Expression Omnibus
MD: Molecular dynamics
MF: Molecular function
MOE: Molecular operating environment
β-MHC: β-myosin heavy chain
NOB: nobilietin
OB: Oral bioavailability
PBS: Phosphate-buffered saline
PPARα: Peroxisome proliferator-activated receptor alpha
PPARγ: Peroxisome proliferator-activated receptor gamma
PE: Phenylephrine
PPI: Protein–protein interactions
PILP: Protein–ligand Interaction Profiler
qPCR: Quantitative real-time-PCR
RMSD: Root mean square deviation
RMSF: Root mean square fluctuation
ROS: Reactive oxygen species
SRplot: Science and Research online plot
TCMSP: The Traditional Chinese Medicine Systematic Pharmacology Database
YASARA: Yet Another Scientific Artificial Reality Application

## References

[B1] Angola B. Botswana B. F. Burundi C. V. Cameroon (2023). World health statistics 2023: monitoring health for the SDGs. Sustain. Dev. Goals.

[B2] Bai Y. Zheng J.-P. Lu F. Zhang X.-L. Sun C.-P. Guo W.-H. (2022). Prevalence, incidence and mortality of hypertrophic cardiomyopathy based on a population cohort of 21.9 million in China. Sci. Rep. 12 (1), 18799. 10.1038/s41598-022-20042-9 36335106 PMC9637201

[B3] Chen J. Zhong J. Wang L.-l. Chen Y.-y. (2021). Mitochondrial transfer in cardiovascular disease: from mechanisms to therapeutic implications. Front. Cardiovasc. Med. 8, 771298. 10.3389/fcvm.2021.771298 34901230 PMC8661009

[B4] Cornell W. D. Cieplak P. Bayly C. I. Gould I. R. Merz K. M. Ferguson D. M. (1996). A second generation force field for the simulation of proteins, nucleic acids, and organic molecules. J. Am. Chem. Soc. 117 (19), 5179–5197. 10.1021/ja955032e

[B5] Da Dalt L. Cabodevilla A. G. Goldberg I. J. Norata G. D. (2023). Cardiac lipid metabolism, mitochondrial function, and heart failure. Cardiovasc Res. 119 (10), 1905–1914. 10.1093/cvr/cvad100 37392421 PMC10681665

[B6] Dungu J. N. Hardy-Wallace A. Dimarco A. D. Savage H. O. (2024). Hypertrophic cardiomyopathy. Curr. Heart Fail Rep. 21 (4), 428–438. 10.1007/s11897-024-00654-0 38488965

[B7] Fougerat A. Bruse J. Polizzi A. Montagner A. Guillou H. Wahli W. (2024). Lipid sensing by PPARα: role in controlling hepatocyte gene regulatory networks and the metabolic response to fasting. Prog. Lipid Res. 96, 101303. 10.1016/j.plipres.2024.101303 39521352

[B8] Gan X. Shu Z. Yan D. Li J. Ofaim S. Albert R. (2023). Network medicine framework reveals generic herbsymptom effectiveness of traditional Chinese medicine. Sci. Adv. 9, eadh0215. 10.1126/sciadv.adh0215 37889962 PMC10610911

[B9] Grevengoed T. J. Klett E. L. Coleman R. A. (2014). Acyl-CoA metabolism and partitioning. Annu. Rev. Nutr. 34 (1), 1–30. 10.1146/annurev-nutr-071813-105541 24819326 PMC5881898

[B10] Han Y. H. Moon H. J. You B. R. Park W. H. (2009). The effect of MG132, a proteasome inhibitor on HeLa cells in relation to cell growth, reactive oxygen species and GSH. Oncol. Rep. 22 (1), 215–221. 10.3892/or_00000427 19513526

[B11] Huang Y. Li W. Sun H. Guo X. Zhou Y. Liu J. (2024). Mitochondrial transfer in the progression and treatment of cardiac disease. Life Sci. 358, 123119. 10.1016/j.lfs.2024.123119 39395616

[B12] Jain S. Pulikuri S. Zhu Y. Qi C. Kanwar Y. S. Yeldandi A. V. (1998). Differential expression of the peroxisome proliferator-activated receptor gamma (PPARgamma) and its coactivators steroid receptor coactivator-1 and PPAR-binding protein PBP in the brown fat, urinary bladder, colon, and breast of the mouse. Am. J. Pathol. 153 (2), 349–354. 10.1016/s0002-9440(10)65577-0 9708794 PMC1852994

[B13] Ke Z. Fan C. Li J. Wang L. Li H. Tian W. (2023). Nobiletin Intake attenuates hepatic lipid profiling and oxidative stress in HFD-Induced nonalcoholic-fatty-liver-disease mice. Molecules 28 (6), 2570. 10.3390/molecules28062570 36985541 PMC10054910

[B14] Koo Y. E. Song J. Bae S. (2018). Use of plant and herb derived medicine for therapeutic usage in cardiology. Medicines 5 (2), 38. 10.3390/medicines5020038 29690545 PMC6023439

[B15] Krieger E. Koraimann G. Vriend G. (2002). Increasing the precision of comparative models with YASARA NOVA--a self-parameterizing force field. Proteins 47 (3), 393–402. 10.1002/prot.10104 11948792

[B16] Krieger E. Vriend G. (2015). New ways to boost molecular dynamics simulations. J. Comput. Chem. 36 (13), 996–1007. 10.1002/jcc.23899 25824339 PMC6680170

[B17] Legchenko E. Chouvarine P. Borchert P. Fernandez-Gonzalez A. Snay E. Meier M. (2018). PPARγ agonist pioglitazone reverses pulmonary hypertension and prevents right heart failure via fatty acid oxidation. Sci. Transl. Med. 10, eaao0303. 10.1126/scitranslmed.aao0303 29695452

[B18] Liu E. H. Zhao P. Duan L. Zheng G.-D. Guo L. Yang H. (2013). Simultaneous determination of six bioactive flavonoids in Citri Reticulatae Pericarpium by rapid resolution liquid chromatography coupled with triple quadrupole electrospray tandem mass spectrometry. Food Chem. 141 (4), 3977–3983. 10.1016/j.foodchem.2013.06.077 23993574

[B19] Mirza A. Z. Althagafi I. I. Shamshad H. (2019). Role of PPAR receptor in different diseases and their ligands: physiological importance and clinical implications. Eur. J. Med. Chem. 166, 502–513. 10.1016/j.ejmech.2019.01.067 30739829

[B20] Montminy M. R. Gonzalez G. A. Yamamoto K. K. (1990). Regulation of cAMP-inducible genes by CREB. Trends Neurosci. 13 (5), 184–188. 10.1016/0166-2236(90)90045-c 1693237

[B21] Murray C. J. L. (2022). The Global Burden of Disease Study at 30 years. Nat. Med. 28 (10), 2019–2026. 10.1038/s41591-022-01990-1 36216939

[B22] Nakagawa Y. Nishikimi T. Kuwahara K. (2019). Atrial and brain natriuretic peptides: hormones secreted from the heart. Peptides 111, 18–25. 10.1016/j.peptides.2018.05.012 29859763

[B23] Noordali H. Loudon B. L. Frenneaux M. P. Madhani M. (2018). Cardiac metabolism — a promising therapeutic target for heart failure. Pharmacol. Ther. 182, 95–114. 10.1016/j.pharmthera.2017.08.001 28821397

[B24] Pan J. Meng L. Li R. Wang Z. Yuan W. Li Y. (2024). Naringenin protects against septic cardiomyopathy in mice by targeting HIF-1α. Biochem. Biophys. Res. Commun. 704, 149613. 10.1016/j.bbrc.2024.149613 38387325

[B25] Previs M. J. O’Leary T. S. Morley M. P. Palmer B. M. LeWinter M. Yob J. M. (2022). Defects in the proteome and metabolome in human hypertrophic cardiomyopathy. Circ.:Heart Fail. 15 (6), e009521. 10.1161/circheartfailure.121.009521 35543134 PMC9708114

[B26] Ramakrishnan V. Pace B. S. (2011). Regulation of γ-globin gene expression involves signaling through the p38 MAPK/CREB1 pathway. Blood Cells Mol. Dis. 47 (1), 12–22. 10.1016/j.bcmd.2011.03.003 21497119 PMC3695476

[B27] Ranjbarvaziri S. Kooiker K. B. Ellenberger M. Fajardo G. Zhao M. Roest A. S. V. (2021). Altered cardiac energetics and mitochondrial dysfunction in hypertrophic cardiomyopathy. Circulation 144 (21), 1714–1731. 10.1161/circulationaha.121.053575 34672721 PMC8608736

[B28] Rubio-Ruíz M. E. Plata-Corona J. C. Soria-Castro E. Díaz-Juárez J. A. Sánchez-Aguilar M. (2024). Pleiotropic effects of peroxisome proliferator-activated receptor alpha and gamma agonists on myocardial damage: molecular mechanisms and clinical evidence—A narrative review. Cells 13 (17), 1488. 10.3390/cells13171488 39273057 PMC11394383

[B29] Schlittler M. Pramstaller P. P. Rossini A. De Bortoli M. (2023). Myocardial fibrosis in hypertrophic cardiomyopathy: a perspective from fibroblasts. Int. J. Mol. Sci. 24 (19), 14845. 10.3390/ijms241914845 37834293 PMC10573356

[B30] Sebastian S. A. Panthangi V. Singh K. Rayaroth S. Gupta A. Shantharam D. (2023). Hypertrophic cardiomyopathy: current treatment and future options. Curr. Probl. Cardiol. 48 (4), 101552. 10.1016/j.cpcardiol.2022.101552 36529236

[B31] Shorbagi M. Fayek N. M. Shao P. Farag M. A. (2022). Citrus reticulata blanco (the common mandarin) fruit: an updated review of its bioactive, extraction types, food quality, therapeutic merits, and bio-waste valorization practices to maximize its economic value. Food Biosci. 47, 101699. 10.1016/j.fbio.2022.101699

[B32] Wan J. Zhang Z. Wu C. Tian S. Zang Y. Jin G. (2023). Astragaloside IV derivative HHQ16 ameliorates infarction-induced hypertrophy and heart failure through degradation of lncRNA4012/9456. Signal Transduct. Target. Ther. 8 (1), 414. 10.1038/s41392-023-01660-9 37857609 PMC10587311

[B33] Wang D. Gao F. Hu F. Wu J. (2022a). Nobiletin alleviates astrocyte activation and oxidative stress induced by hypoxia In Vitro. Molecules 27 (6), 1962. 10.3390/molecules27061962 35335325 PMC8953234

[B34] Wang L. Cai Y. Jian L. Cheung C. W. Zhang L. Xia Z. (2021). Impact of peroxisome proliferator-activated receptor-α on diabetic cardiomyopathy. Cardiovasc. Diabetol. 20 (1), 2. 10.1186/s12933-020-01188-0 33397369 PMC7783984

[B35] Wang W. Wang J. Yao K. Wang S. Nie M. Zhao Y. (2022b). Metabolic characterization of hypertrophic cardiomyopathy in human heart. Nat. Cardiovasc. Res. 1 (5), 445–461. 10.1038/s44161-022-00057-1 39195941

[B36] Wang X. Ma Y. Xu Q. Shikov A. N. Pozharitskaya O. N. Flisyuk E. V. (2023). Flavonoids and saponins: what have we got or missed? Phytomedicine 109, 154580. 10.1016/j.phymed.2022.154580 36610132

[B37] Wijnker P. J. M. Dinani R. van der Laan N. C. Algül S. Knollmann B. C. Verkerk A. O. (2024). Hypertrophic cardiomyopathy dysfunction mimicked in human engineered heart tissue and improved by sodium–glucose cotransporter 2 inhibitors. Cardiovasc. Res. 120 (3), 301–317. 10.1093/cvr/cvae004 38240646 PMC10939456

[B38] Wu X. Zheng D. Qin Y. Liu Z. Zhang G. Zhu X. (2017). Nobiletin attenuates adverse cardiac remodeling after acute myocardial infarction in rats via restoring autophagy flux. Biochem. Biophys. Res. Commun. 492 (2), 262–268. 10.1016/j.bbrc.2017.08.064 28830813

[B39] Yahya S. A. Al-Shawi N. N. (2024). Hepatoprotective effect of nobiletin against 5-fluorouracil induce hepatotoxicity. Curr. Res. Pharmacol. Drug Discov. 7, 100199. 10.1016/j.crphar.2024.100199 39411523 PMC11474214

[B40] Yin Y. Tang D. Chen M. Huang X. Zhang G. Zeng L. (2023). SRplot: a free online platform for data visualization and graphing. PLoS One 18 (11), e0294236. 10.1371/journal.pone.0294236 37943830 PMC10635526

[B41] Yin Y. Yuan H. Wang C. Pattabiraman N. Rao M. Pestell R. G. (2006). 3-Phosphoinositide-Dependent protein Kinase-1 activates the peroxisome proliferator-activated Receptor-γ and promotes adipocyte differentiation. Mol. Endocrinol. 20 (2), 268–278. 10.1210/me.2005-0197 16150867

[B42] Yu X. Sun S. Guo Y. Liu Y. Yang D. Li G. (2018). Citri reticulatae pericarpium (Chenpi): Botany, ethnopharmacology, phytochemistry, and pharmacology of a frequently used traditional Chinese medicine. J. Ethnopharmacol. 220, 265–282. 10.1016/j.jep.2018.03.031 29628291

[B43] Zhang J. Qiu H. Huang J. Ding S. Huang B. Zhou P. (2019). EETs/PPARs activation together mediates the preventive effect of naringenin in high glucose-induced cardiomyocyte hypertrophy. Biomed. Pharmacother. 109, 1498–1505. 10.1016/j.biopha.2018.10.176 30551401

[B44] Zhang N. Wen-Ying W. Yang Z. Che Y. Jin Y.-G. Liao H.-H. (2017). Nobiletin, a polymethoxy flavonoid, protects against cardiac hypertrophy induced by pressure-overload via inhibition of NAPDH oxidases and endoplasmic reticulum stress. Cell. Physiol. Biochem. 42 (4), 1313–1325. 10.1159/000478960 28700997

[B48] Zhang Q. Luo P. Chen J. Yang C. Xia F. Zhang J. (2022). Dissection of targeting molecular mechanisms of aristolochic acid-induced nephrotoxicity via a combined deconvolution strategy of chemoproteomics and metabolomics. Int. J. Biol. Sci. 18 (5), 2003–2017. 10.7150/ijbs.69618 35342337 PMC8935225

[B45] Zhou L. Gu W. Kui F. Gao F. Niu Y. Li W. (2021). The mechanism and candidate compounds of aged citrus peel (chenpi) preventing chronic obstructive pulmonary disease and its progression to lung cancer. Food Nutr. Res. 65. 10.29219/fnr.v65.7526 PMC8254466 34262419

[B46] Zou J. Wang J. Ye W. Lu J. Li C. Zhang D. (2022). Citri reticulatae pericarpium (Chenpi): a multi-efficacy pericarp in treating cardiovascular diseases. Biomed. Pharmacother. 154, 113626. 10.1016/j.biopha.2022.113626 36058153

[B47] Zou Y. Song L. Wang Z. Ma A. Liu T. Gu H. (2004). Prevalence of idiopathic hypertrophic cardiomyopathy in China: a population-based echocardiographic analysis of 8080 adults. Am. J. Med. 116(1):14–18. 10.1016/j.amjmed.2003.05.009 14706660

