# Supplementary material for: Nobiletin protected against hypertrophic cardiomyopathy via targeting PPARα
Source: Front Pharmacol. 2025 Aug 4;16:1628625. doi: 10.3389/fphar.2025.1628625 (PMC12358417; doi:10.3389/fphar.2025.1628625)
Supplement: Supplementary file 1 [file DataSheet1.pdf]

Supplementary material

Table S1. Potential Targets of *Citrus reticulata* active ingredients

Gene name	Gene Symbol	Uniprot ID	Gene name	Gene Symbol	Uniprot ID
3-hydroxy-3-methylglutaryl-coenzyme A reductase	HMGCR	P04035	Catalase	CAT	P04040
4-aminobutyrate aminotransferase, mitochondrial	ABAT	X5D8S1	Cellular tumor antigen p53	TP53	Q12888
Adiponectin	ADIPOQ	Q15848	Coagulation factor VII	F7	P08709
Aldo-keto reductase family 1 member C1	AKR1C1	Q04828	Cyclic AMP-responsive element-binding protein 1	CREB1	P16220
Androgen receptor	AR	P10275	Cytochrome P450 19A1	CYP19A1	P11511
Apolipoprotein B-100	APOB	P04114	Cytosolic phospholipase A2	PLA2G4A	P47712
Apoptosis regulator BAX	BAX	Q07812	Dipeptidyl peptidase IV	DPP4	P27487
Apoptosis regulator Bcl-2	BCL2	P10415	DNA topoisomerase II	TOP2B	Q02880
Aspartate aminotransferase, cytoplasmic	GOT1	P17174	Ephrin type-B receptor 2	EPHB2	P29323
Bcl2 antagonist of cell death	BAD	Q92934	Estrogen receptor	ESR1	P03372
Beta-lactamase	LACTBL1	A8MY62	Estrogen receptor beta	ESR2	Q92731
Calcium-activated potassium channel subunit alpha 1	KCNMA1	Q12791	Fatty acid synthase	FASN	A0A0U1RQF0
Calmodulin	CALM2	P0DP24	Glutathione reductase, mitochondrial	GSR	P00390
Caspase-3	CASP3	P42574	Glutathione S-transferase P	GSTP1	P09211

Caspase-9	CASP9	P55211	Glycogen synthase kinase-3 beta	GSK3B	P49841
Low-density lipoprotein receptor	LDLR	P01130	Heat shock protein HSP 90	HSP90AA1	P07901
Matrix metalloproteinase-9	MMP9	P14780	Liver carboxylesterase 1	CES1	Q9NZK5
Metalloproteinase inhibitor 1	TIMP1	P01033	Nuclear receptor coactivator 2	NCOA2	Q15596
Microsomal triglyceride transfer protein large subunit	MTTP	P55157	Peroxisome proliferator-activated receptor alpha	PPARA	Q07869
Mineralocorticoid receptor	NR3C2	P08235	Peroxisome proliferator-activated receptor gamma	PPARG	P37231
Mitogen-activated protein kinase 1	MAPK1	P28482	Phosphatidylinositol-4,5-bisphosphate 3-kinase catalytic subunit, gamma isoform	PIK3CG	P48736
Mitogen-activated protein kinase 3	MAPK3	Q16644	Phospholipase B1, membrane-associated	PLB1	Q6P1J6
Mitogen-activated protein kinase 8	MAPK8	P45983	Potassium voltage-gated channel subfamily H member 2	KCNH2	Q12809
mRNA of Protein-tyrosine phosphatase, non-receptor type 1	PTPN1	P18031	Progesterone receptor	PGR	P06401
Multidrug resistance-associated protein 1	ABCC1	P33527	Prostaglandin G/H synthase 1	PTGS1	P23219
Nitric oxide synthase, inducible	NOS2	P35228	Prostaglandin G/H synthase 2	PTGS2	P35354
Nuclear receptor coactivator 1	NCOA1	Q15788	RAC-alpha serine/threonine-protein kinase	AKT1	P31750

Nuclear receptor coactivator 2	NCOA2	Q15596	Scavenger receptor cysteine-rich type 1 protein M130	CD163	Q86VB 7
Peroxisome proliferator-activated receptor alpha	PPARA	Q07869	Serine/threonine-protein kinase Chk1	CHEK1	O14757
Peroxisome proliferator-activated receptor gamma	PPARG	P37231	Sodium channel protein type 5 subunit alpha	SCN5A	Q14524
Phosphatidylinositol-4,5-bisphosphate 3-kinase catalytic subunit, gamma isoform	PIK3CG	P48736	Sterol O-acyltransferase 1	SOAT1	P35610
Phospholipase B1, membrane-associated	PLB1	Q6P1J6	Sterol O-acyltransferase 2	SOAT2	O75908
Potassium voltage-gated channel subfamily H member 2	KCNH2	Q12809	Sterol regulatory element-binding protein 1	SREBF1	P36956
Progesterone receptor	PGR	P06401	Superoxide dismutase [Cu-Zn]	SOD1	P00441
Prostaglandin G/H synthase 1	PTGS1	P23219	Thrombin	SERPIND1	P05546
Prostaglandin G/H synthase 2	PTGS2	P35354	Transcription factor AP-1	JUN	P05412
RAC-alpha serine/threonine-protein kinase	AKT1	P31749	Transcription factor p65	RELA	Q04206
Scavenger receptor cysteine-rich type 1 protein M130	CD163	Q86VB 7	Trypsin-1	PRSS1	P07477
Serine/threonine-protein kinase Chk1	CHEK1	O14757	UDP-glucuronosyltransferase 1-1	UGT1A8	Q9HAW 9
Sodium channel protein type 5 subunit alpha	SCN5A	Q14524	Sterol regulatory element-binding protein 1	SREBF1	P36956
Sterol O-acyltransferase 1	SOAT1	P35610	Superoxide dismutase [Cu-Zn]	SOD1	P00441
Sterol O-acyltransferase 2	SOAT2	O75908	Thrombin	SERPIND1	P05546
Transcription factor AP-1	JUN	P05412	Trypsin-1	PRSS1	P07477

Transcription factor p65	RELA	Q04206	UDP-glucuronosyltransferase 1-1	UGT1A8	Q9HAW9

Table S2. Total of DEGs of ups

DEGs of ups								
IRX2	KIF13B	SPTLC2	FLJ30277	MAPK12	PANK1	PRR16	PLCE1	TNRC6A
PROS1	INHBE	FLJ39575	NUDT16	FSTL3	REST	TLL2	SORT1	LEPREL1
PPP1R16B	N4BP2	PIGZ	PCDH7	PYGB	CBLC	DIO2	ZBTB47	KCNJ5
PPP2R2B	ALDH4A1	GABRB1	LAD1	ATP1A2	KCNH7	MAF	EFCBP1	KIAA1202
SLITRK4	ESRRB	MAPK8IP1	SFRP1	MYO16	MAP3K7IP1	YTHDC1	MYOZ3	PER2
SNCA	ANKRD15	CSTF1	TCEAL2	TNNT1	IL2	TNRC6B	SNCA	METT10D
HSPA2	TPO	NAP1L3	PPARA	C14orf126	LOC402176	ZNF662	MUM1L1	USP48
NTNG2	SIN3B	AZIN1	COLQ	STX19	WDR31	APOA1	GLDN	SOD3
C1orf142	CFL2	VPS13A	PDAP1	C10orf46	PIK3R1	MYST4	C4orf29	CLDND2
LRFN3	LOC283932	ABAT	LTBP2	LRRC1	LMX1B	ORMDL3	CTNNA3	MRPL43
SLC16A5	EDARADD	NUFIP1	YIPF7	LCN12	S100A13	TEF	C7orf41	FLJ45717
MMP23A	KIAA0319L	CRAMP1L	TAOK2	MFI2	PPFIA4	ZNF33A	TPM3	SNAPC1
C14orf132	PDDC1	IPO8	ENAM	GUCA1C	DENND1A	VGLL2	IGF2BP3	SCN2B
SLC1A7	ZMYND17	COL7A1	MYO18B	CHRM2	ZNF740	SBK1	MITF	COL21A1
RASL11B	HSPG2	DBC1	PDE6B	HEPH	FGF1	PLEKHA4	SCRN1	ZFP2
ACE2	FMOD	SMARCD1	PACSIN1	COL23A1	WDR66	OSBPL1A	NES	GRK4
CTNND2	TGM2	CCDC86	CRTAM	KLHL34	ODF2	RFC2	VTN	FLJ20297

TRIM69	FGF8	PER3	CAPN12	HDAC5	SPPL2B	DKFZp451A211	PRRX1	KIF13A
USP46	VIPR2	SEMA6D	ST8SIA5	DDR1	NPPA	GSTM3	TNMD	DNASE1L3
JAK2	CNN1	C10orf65	BDNF	FLJ22028	ITGA10	SETBP1	ARHGAP24	POLL
FZD3	PAK1	C21orf56	GPC1	FSCN2	PKLR	C10orf83	DKFZp686O24166	POSTN
FCMD	DYRK1B	MAP1A	ZNF764	KLHL26	ZBTB16	BCOR	DIXDC1	LOC221442
TMEM74	ZBTB16	C10orf67	UCHL1	C2orf27	HIF3A	KCNMB2	SOLH	ZMAT1
FBXO9	FLJ43806	C10orf71	CTAGE5	CRIM1	PPFIA1	HMBOX1	KIAA1772	AXIN2
TPM3	IQCE	PAQR8	IRX6	MLLT11	C5orf23	SUPT3H	SLC9A8	ENAH
CASQ1	FGF18	KCNA4	CAPN3	ST8SIA2	SH3GL2	HFE2	PRRT3	CYP26B1
JPH2	C10orf30	CLK3	DCAMKL2	TMEM139	LOC387758	IGSF1	RNF165	OBSCN
RASAL2	PKD1	DNAJC18	MT1M	LOC196549	RGS17	KIAA1026	ZNF785	KCNC4
ZNF577	FBXO38	PCDHGC3	JUB	KLHDC6	SALL2	PLEKHA7	NCAM1	ZFP28
NMNAT2	DDAH1	KIAA1244	DNAH1	GDF3	ARNT2	SLA/LP	KCNK6	KIAA0652
CXXC4	ERCC4	ENO2	GPR63	LOC147804	ARHGAP24	IGFBP5	YWHAE	WDR24
WNT2B	ARHGAP24	HK1	C21orf7					

Table S3. Total of DEGs of downs

Sig_Down								
C10orf11	ART5	PLEK	AKAP3	CD86	SLC2A5	NKD2	C8orf4	SLA
C1orf105	LY96	HOXB2	TIMM8B	C17orf58	NTF3	RGS10	BTLA	CFD
MYH6	RASGEF1B	ADORA3	SLC16A14	RSAD2	C3	TCF21	FEZF2	AGTR1
OGFRL1	GNB4	NCR3	TUBA3E	PLSCR1	TMEM155	SLC9A9	BAIAP2L1	APOB
NR4A2	ABCB1	HMHA1	MGST1	DOCK2	ABCA9	D4S234E	SLC1A5	FGD3
RASD1	FLJ25680	WNT2	LAPTM5	LY9	LRRC8C	LSAMP	POF1B	ZWINT
C21orf93	CXCL10	KLRB1	CLEC10A	ANKRD37	CD38	C20orf46	CD68	BMP5
HSPA1B	NGFRAP1L1	BTNL3	MSC	SLC2A3	SGOL2	ZFP36	CCL2	LCP1
ZNF8	ATF3	SOX11	MAFB	CD163L1	CEBPD	CEL	UNQ6411	BOLL
HLA-DRB3	TNFAIP8L3	MGAT4C	RHAG	SLC2A13	MS4A7	RHPN2	BHLHB5	CXCL2
METTL7B	SNX10	FLJ40288	INCENP	NEDD9	NCF4	IGSF3	DPEP2	AQP12A
AGXT2L1	CORIN	AXUD1	MGC45491	STMN1	EPHA8	LCP2	EGFR	BAI3
ABCG1	MCTP1	HCST	HNMT	AMICA1	RUNX3	CASP1	TTYH2	CHRD
FABP6	KITLG	OCIAD2	IL32	CCR2	TCF21	DKFZP564J0863	SKAP1	ELA2
SMTNL2	FAM63A	ARHGAP25	ALDH3B2	DDX3Y	LOC387934	RGS16	MGC34824	PLAU
ENPEP	UBXD3	SNHG3-RCC1	MS4A6A	CHML	GNA15	ELF4	TFPI	STARD5
CPNE4	HIST1H2BC	NP	FOS	F13A1	SUSD4	DACH1	PELI1	PSORS1C1
DOCK10	SNF1LK	HOXC6	MT3	SLC35D1	EFHC2	DOCK8	GOT1L1	SUSD1
CPA3	IKIP	CCDC109B	FGF17	GNLY	POLE2	MTP18	F5	BBS9
TCTEX1D1	TAS2R4	CLIC3	CACHD1	GAS2L3	ISG20	MYB	NEDD9	CD209
OBFC2A	FAM81A	SPOCK1	NR3C1	FAM43A	SLC9A3R1	FGL2	KLK5	FCGR2A
DUSP1	CTSC	CLCC1	KLF4	GNA15	SUSD4	IL1R1	SAMSN1	CDC7

PRIM2A	DUSP2	PDGFRA	CLDN23	PCP4	GBX2	OR52E6	WDR89	RAC2
DEFB121	PCDH18	VAMP8	C4orf31	ST8SIA4	C1R	RUNX3	TCF21	LOC387934
CACNA2D4	CNTNAP3	C6orf204	RNF125	STC2	FCN3			

Table S4. Hydrogen bonds of PPARγ with the main active ingredients of *C. Reticulata* from docking analysis

Protein	3U9Q-PPARγ			
ligand	type	AA	D-A	angle
Nobiletin	H-BOND	SER-342	3.12	164.4
	H-BOND	ILE-262	3.93	158.22
Naringenin	H-BOND	HIS-449	3.19	121.79
	H-BOND	TYR-473	4.02	110.32
	H-BOND	HIS-323	3.67	114.88

Table S5. Non - covalent interactions of PPARα with the main active ingredients of *C. Reticulata*

Protein	PPARα (7BQ2)			
ligand	type	AA	D-A	angle
Nobiletin	Hydrophobic	THR-279	3.53	-
	Interactions	LEU-321	3.49	-
		VAL-332	3.49	-
	Hydrogen Bonds	ALA-333	2.91	147.94
Naringenin	Hydrophobic	THR-279	3.44	-
	Interactions	LEU-321	3.87	-
		VAL-324	3.86	-
	Hydrogen Bonds	ASN-219	3.23	124.55
		MET-220	3.83	106.45
		SER-323	3.67	120.31

Table S6. Non - covalent interactions of PPARγ with the main active ingredients of *C. Reticulata*

Protein	PPARγ (3U9Q)			
ligand	type	AA	D-A	angle
Nobiletin	Hydrophobic Interactions	ANG-288	3.70	-
		LEU-330	3.90	-
	Hydrogen Bonds	ILE-262	3.92	158.22
		SER-324	3.12	164.4
Naringenin	Hydrophobic Interactions	ILE-281	3.7	-
		PHE-282	3.54	-
		PHE-282	3.69	-
		ILE-326	3.97	-
		TYR-327	3.67	-
		LEU-353	3.76	-
		PHE-363	3.33	-
		PHE-363	3.62	-
	Hydrogen Bonds	HIS-323	3.67	144.88
		HIS-449	3.19	121.79
		TYR-473	4.02	110.32

Table S7. Non - covalent interactions of CREB1 with the main active ingredients of *C. Reticulata*

Protein	CREB1 (5ZK1)			
ligand	type	AA	D-A	angle
Nobiletin	Hydrophobic Interactions	ARG-20	3.89	-
		LYS-21	3.96	-
		PHE-22	3.54	-
	Hydrogen Bonds	ARG-20	3.62	161.36
		LYS-21	3.09	143.20
		LYS-303	3.95	146.88
	π-Stacking	PHE-22	5.23	72.83
	π-Cation Interactions	ARG-20	4.27	-
Naringenin	Hydrophobic Interactions	ARG-301	3.99	-
		ARG-302	3.56	-
		ARG-302	3.69	-
		GLU-306	3.82	-
	Hydrogen Bonds	LYS-305	3.19	144.45
		GLU-306	3.40	154.72
		LYS-309	2.97	128.71

Supplementary Figures

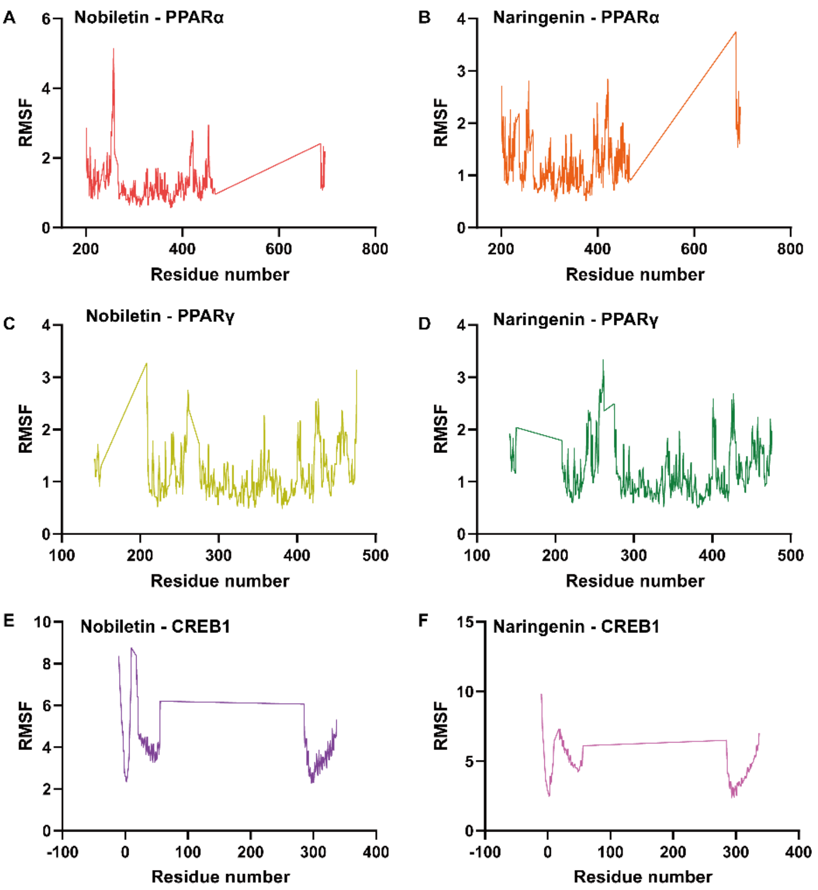

Figure 1. The root mean square fluctuation (RMSF) analysis to assess the stability of amino acid molecules. (A, C and E) RMSF analysis results of nobiletin with PPARα, PPARγ and CREB1. (B, D and F) RMSF analysis results of nobiletin with PPARα, PPARγ and CREB1.
